# Cladribine Preserves Normal Central Nervous System Cellular Activity and Promotes Neuroprotection to Oxidative Stress Damage

**DOI:** 10.3390/ijms262311311

**Published:** 2025-11-22

**Authors:** Herena Eixarch, Laura Calvo-Barreiro, Nicolás Fissolo, Ursula Boschert, Arnau Hervera, Manuel Comabella, Xavier Montalban, Carmen Espejo

**Affiliations:** 1 Servei de Neurologia, Centre d’Esclerosi Múltiple de Catalunya (Cemcat), Institut de Recerca Vall d’Hebron (VHIR), Hospital Universitari Vall d’Hebron, 08035 Barcelona, Spain; lac4018@med.cornell.edu (L.C.-B.); nicolas.fissolo@vhir.org (N.F.); arnau.hervera@vhir.org (A.H.); manuel.comabella@vhir.org (M.C.); xavier.montalban@cem-cat.org (X.M.); 2 Universitat Autònoma de Barcelona, 08193 Bellaterra, Spain; 3 Centro de Investigación Biomédica en Red de Enfermedades Neurodegenerativas (CIBERNED), Instituto de Salud Carlos III, Ministerio de Ciencia, Innovación y Universidades, 28031 Madrid, Spain; 4 Merck Healthcare KGaA, F135/002, 64293 Darmstadt, Germany; ursula.boschert@external.merckgroup.com

**Keywords:** cladribine, multiple sclerosis, disease modifying treatment, central nervous system, immunomodulation, neuroprotection, neurodegeneration

## Abstract

Multiple sclerosis (MS) is a chronic neuroinflammatory and demyelinating disease that causes disability in patients. Cladribine is an oral treatment that is used in relapsing–remitting and active secondary progressive MS. T and B lymphocytes are especially sensitive to cladribine, which are transiently depleted upon short treatment courses. However, cladribine crosses the blood–brain barrier (BBB), supporting the hypothesis that cladribine may affect central nervous system (CNS)-resident cells. In this study, we used human primary cells and human cell lines to test the effect of cladribine, at therapeutic concentrations, on cells of the CNS. In these conditions, cladribine did not affect survival, proliferation and the capacity of producing cytokines of human microglial cells (HMC3 cell line) or primary human astrocytes but enhanced the production of oxygen reactive species in both cell types. The initial differentiation of primary human neuronal progenitor cells was impaired when continuously exposed to the maximum therapeutic concentration of cladribine, but not when lower concentrations were used. However, cladribine protected differentiated SH-SY5Y human neuroblastoma cell line from oxidative stress-related cell death. In conclusion, using different in vitro cell models, we demonstrate that cladribine maintains the normal function of CNS glia and protects neuronal cells from oxidative stress damage.

## 1. Introduction

Multiple sclerosis (MS) is an immune-mediated disease of autoimmune origin that affects the central nervous system (CNS), leading to inflammation, formation of demyelination plaques and subsequent neurodegeneration that causes disability in patients [1,2]. MS affects 2.8 million people worldwide [3] and is the first cause of non-traumatic disability in young adults. The etiology of MS remains unclear; however, a combination of genetic predisposition and environmental factors has been identified as critical in triggering disease onset [4,5]. The immunopathogenesis of the disease has been linked to CD4+ T cells based on findings from the widely used experimental autoimmune encephalomyelitis (EAE) animal model [6] and from studies with MS samples [7,8]. In addition to CD4+ T cells, demyelinated areas in MS, also known as lesions, are infiltrated by a variety of immune cells including CD8+ T cells, B cells, and macrophages. This diverse cellular presence highlights the complexity of the immune response involved in MS pathogenesis [9]. Notably, evidence from anti-CD20 therapies has underscored the pivotal role of B cells in MS for their antigen-presenting capacity and pro-inflammatory cytokine secretion [10]. Several disease modifying therapies (DMTs) have been approved in recent decades for the treatment of relapsing–remitting MS [11,12], aimed at reducing the frequency of relapses and preventing the accumulation of disability. The efficacy of the approved DMTs has changed the natural history of the disease, reducing the risk of conversion to secondary progressive MS [13]. One of the therapies that contributed to this shift in MS health care is cladribine, which was originally developed for hematological malignancies. The efficacy and safety of cladribine tablets was proved in CLARITY and ORACLE clinical trials [14,15,16,17,18]. It received marketing authorization from the European Medicines Agency (EMA) in 2017 for patients with highly active relapsing MS and in 2019 obtained approval from U.S. Food and Drug Administration (FDA) for relapsing–remitting MS and active secondary progressive MS.

Cladribine (2-chlorodeoxyadeosine, 2-CdA) is a synthetic purine nucleoside analogue of deoxyadenosine that is converted intracellularly into its active triphosphate form through an initial phosphorylation by deoxycytidine kinase (DCK). The active metabolite is incorporated into DNA, inducing replicative stress and impairing DNA repair, ultimately leading to cell apoptosis. The selective cytotoxicity of cladribine towards lymphocytes is attributed to their high DCK-to–5’-nucleotidase (5-NT) ratio, which facilitates intracellular accumulation of the active compound. Consequently, T and B lymphocytes are particularly susceptible to cladribine’s effects [19,20]. In clinical MS use, cladribine tablets are administered orally in two short annual courses, causing transient lymphocyte depletion, followed by immune reconstitution. For instance, recent studies demonstrate that treatment with cladribine tablets induce a shift on the immune system towards a less pathogenic state and reduce neuroinflammation and axonal damage [21,22]. Alternatively, beyond its canonical mechanism of action through DCK-dependent activation, non-phosphorylated cladribine may also exert immunomodulatory effects and promote neurogenesis via adenosine receptor agonism, primarily through A1 receptors, but also through A2a receptors for which cladribine has low affinity [23,24]. A1 receptors are the most widely distributed among adenosine receptors and are expressed across virtually all tissues, with particularly high levels in the CNS [25]. Importantly, A1 adenosine receptors are key regulators of inflammation, promoting anti-inflammatory responses and limiting inflammation-induced tissue damage [26,27], suggesting a potential additional therapeutic mechanism of cladribine relevant to MS pathogenesis.

Cladribine can cross the blood–brain barrier (BBB), with approximately 25% of its plasma concentration detected in the cerebrospinal fluid (CSF) [20,28]. This CNS penetration enables cladribine to interact directly with resident immune cells, astrocytes and microglia, thereby potentially modulating their immunological activity and modifying the local inflammatory environment. Although neural and glial cells exhibit a relatively low DCK/5-NT ratio—potentially limiting cladribine’s cytotoxic activation—the drug may still influence key neuroinflammatory and neurodegenerative pathways, not only in its active form but also as a prodrug through adenosine receptors. In this context, cladribine tablets may influence neurodegenerative and neuroregenerative processes, as well as modulate the response of CNS-resident cells to pathogenic insults in MS pathogenesis.

In the present study we investigated how cladribine, functioning through DCK-dependent or independent pathways, may influence immune–neural interactions within the CNS, providing further insight into its mechanism of action beyond lymphocyte depletion. Using in vitro primary and cell line cultures of human CNS-immune cell (microglia and astrocytes), neural cells and oligodendroglia cells, we studied the effect of active cladribine and non-phosphorylated cladribine on the proliferation, survival and immune function of immune CNS-resident cells, how cladribine potentially interferes with neural and glial differentiation processes and its potential to modulate the cytotoxicity associated with inflammatory, excitotoxic and oxidative stress insults in neuron-like and oligodendroglia-like cells.

## 2. Results

### 2.1. Effect of Cladribine on Human Microglial Cells and Primary Astrocytes

#### 2.1.1. Steady-State Cladribine Concentration Does Not Affect Cell Survival or Proliferation of HMC3 Human Microglia Cell Line and Human Primary Astrocytes

**Figure 1:**
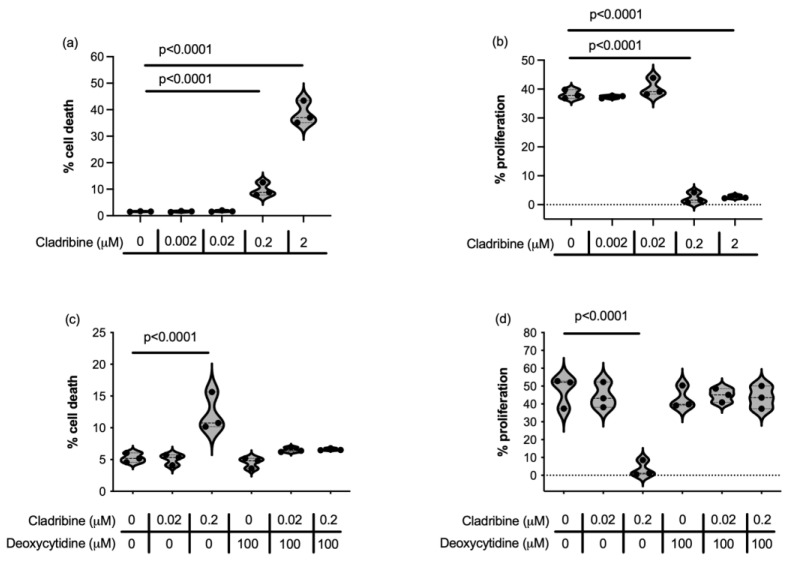
Effect of cladribine on microglial cell survival (**a**,**c**) and proliferation (**b**,**d**) through deoxycytidine kinase (DCK) activity (**a**,**b**) and independently of DCK activity (**c**,**d**). Cell death and proliferation were determined by flow cytometry, using fixable viability stain and Click-iT^®^ Plus EdU flow cytometry assay, respectively. For statistical analysis, data were compared to the basal condition (cells cultured with medium and the vehicle in which cladribine and deoxycytidine were dissolved). Three independent experiments were performed, and each condition was determined in two replicates.

Cladribine can cross the BBB, nonetheless it is estimated that 25% of the concentration found in blood reaches the CNS. Since active cladribine dampens the cell cycle and induces apoptosis in dividing cells, we first examined whether the maximum mean estimated brain concentration of cladribine (0.02 µM) received by MS patients could potentially affect the proliferative capacity and survival of microglial cells and astrocytes. We used an immortalized human microglial cell line [29,30], embryonic microglia clone 3 (HMC3), and primary human astrocytes exposed to increasing concentrations of cladribine. When the drug was principally dependent on DCK activity, toxicity appeared only at high concentrations of cladribine (2 µM, 0.2 µM) in terms of cell death (Figure 1a,c, Supplemental Tables S1 and S3) and proliferation (Figure 1b,d, Supplemental Tables S2 and S4) in HMC3 microglial cells. However, doses reflecting estimated human MS brain exposure levels of the drug (0.02 µM and 0.002 µM) were not toxic for HMC3 cells. Moreover, when DCK activity was saturated by deoxycytidine, so that cladribine could not be phosphorylated to its active form, the toxic effect observed with the high dose of cladribine was completely abolished, demonstrating that cladribine-driven inhibition of survival and proliferation at high doses was dependent on DCK activity (Figure 1c and Figure 1d, respectively, Supplemental Tables S3 and S4).

**Figure 2:**
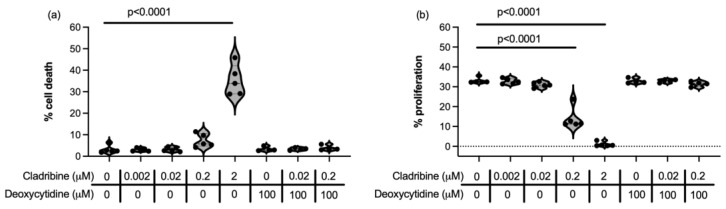
Effect of cladribine in cell death and proliferation in human astrocytes. Cell death (**a**) and proliferation (**b**) were assessed in human astrocytes cultured in the presence of increasing concentrations of cladribine alone or together with deoxycytidine. Cell death and proliferation were determined by flow cytometry, using fixable viability stain and Click-iT^®^ Plus EdU flow cytometry assay, respectively. For statistical analysis, data were compared to the basal condition (cells cultured with medium and vehicle). Data represent 5 independent experiments (4 experiments for deoxycytidine conditions) and each condition was determined in two replicates.

Consistent with these results, cladribine affected primary human astrocyte survival and proliferation only at high doses, and this toxic effect was abolished when DCK was unable to phosphorylate cladribine into its active form (Figure 2a,b, Supplemental Tables S5 and S6). Indeed, human astrocytes exposed to the concentration of cladribine reflecting the mean estimated brain exposure in MS patients presented unaltered survival and capacity to proliferate when cladribine function was dependent or independent on DCK phosphorylation (Figure 2a,b, Supplemental Tables S5 and S6).

Thus, our results show that, 0.02 µM, the maximum concentration of cladribine that reaches the CNS in MS patients is not toxic for microglial cells or astrocytes.

#### 2.1.2. Immune Function of HMC3 Human Microglia Cell Line and Human Primary Astrocytes Exposed to Cladribine

**Figure 3:**
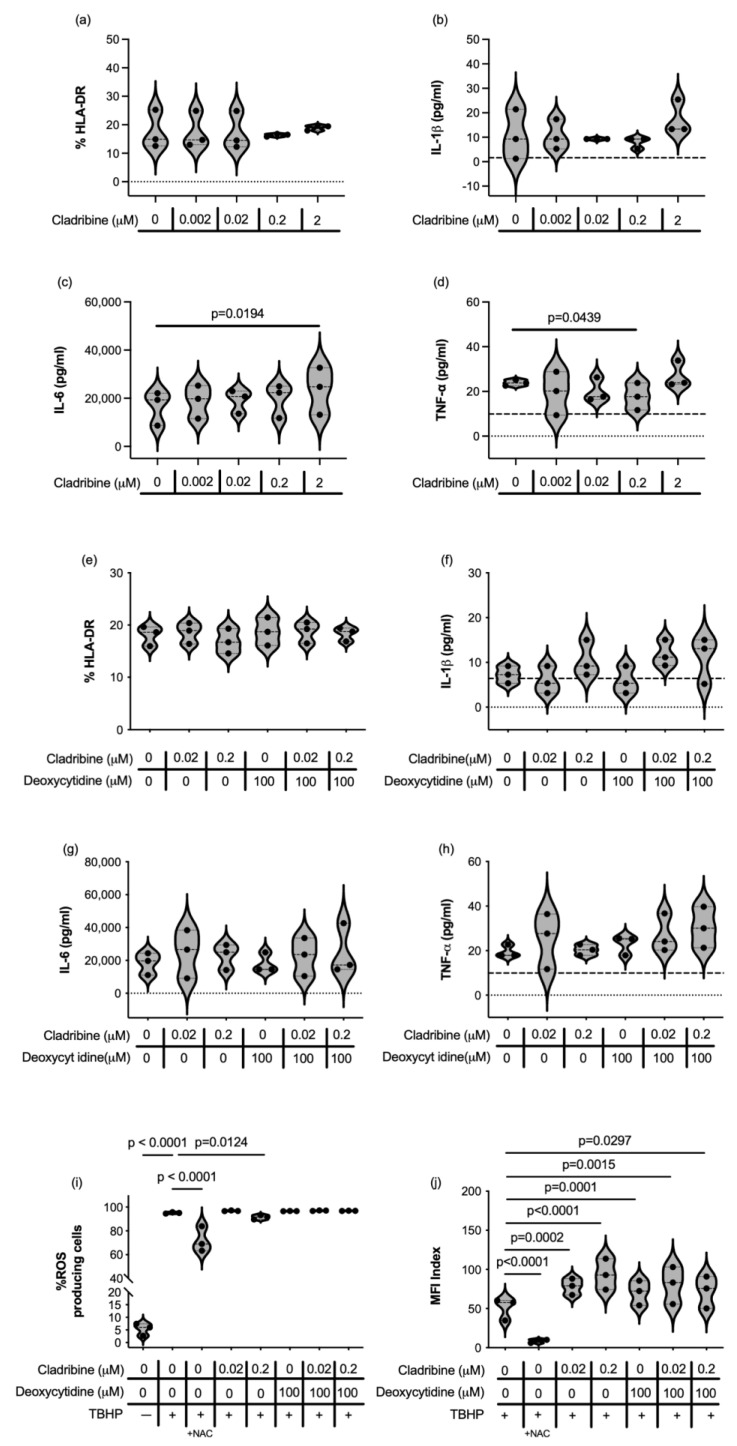
Effect of cladribine on the immune cell function of HMC3 microglial cell line. The effect of cladribine on immune function of microglial cells was assessed upon cytokine stimulation. HLA-DR expression was determined by flow cytometry and cytokine release (IL1-β IL-6 and TNF-α by Procartaplex Immunoassay, and both determinations were assessed in a DCK dependent manner (**a**–**d**) and independently of DCK activity (**e**–**h**). The dashed line in (**b**,**d**,**f**,**h**) indicates the lower limit of quantification of the analytes. Three independent experiments were assessed. For statistical analysis, data were compared to the Stimulated condition (HMC3 cells stimulated with cytokines). ROS production was assessed by means of the percentage of HMC3 microglial cells positive for CellROX by flow cytometry in the different experimental conditions (**i**) and the mean fluorescent intensity (MFI) of each experimental condition respect to the basal values (**j**) (MFI Index). A condition of HMC3 cells stimulated with TBHP and treated with N-acetylcysteine (NAC) was included as a positive control for inhibition of ROS production (indicated in the graphs as +NAC). All experimental conditions were compared to the TBHP condition (cells stimulated with TBHP). Three independent experiments were performed and each condition was determined in two replicates.

Cladribine has also been proposed to have other functions beyond cell cycle arrest and induction of apoptosis, including immunomodulation. This lead us to assess whether the maximum concentration of cladribine that reaches the CNS in the context of MS therapy could somehow affect the immune function of microglial cells and astrocytes. HMC3 cells retain properties of primary microglia and are responsive to stimulus such as lipopolysaccharide (LPS) or interferon (IFN)-γ [30]. In our experimental setting, cytokine-stimulated HMC3 microglial cells upregulated the expression of the major histocompatibility class II molecule HLA-DR (Figure 3a), showed detectable concentrations of interleukin (IL)-1β (Figure 3b), produced high amounts of IL-6 (Figure 3c) and released tumor necrosis factor (TNF)-α (Figure 3d). In terms of cytokine secretion and activation, cladribine only increased IL-6 production at the 2 µM concentration (Figure 3c, Supplemental Table S9) and decreased production of TNF-α at 0.2 µM (Figure 3d, Supplemental Table S10), which are far from the maximum concentration of the drug that reaches the CNS in MS patients, in addition this effect was not consistent across experiments (see Figure 3h, Supplemental Table S14). Overall, cladribine did not impair the immune function of HMC3 microglial cells in either its phosphorylated (Figure 3a–d, Supplemental Tables S7–S10) or non-phosphorylated form (Figure 3e–h, Supplemental Tables S11–S14) at any of the concentrations tested.

Increased reactive oxygen species (ROS) production by activated microglia is implicated in the pathogenesis of MS, contributing to oxidative stress neuronal damage and death. On the other hand, cellular stress due to external insults or metabolic disturbances can trigger an imbalance in ROS production, leading to oxidative stress and subsequent cellular damage and disruption of normal cell function. Almost all microglial cells produced ROS after tert-butyl hydroperoxide (TBHP) stimulation, and the mean estimated brain concentration of cladribie (0.02 μM) did not exert a relevant biological effect in terms of reducing the percentage of cells producing ROS, although 0.2 μM high dose slightly decreased the frequency of HMC3 producing ROS (Figure 3i, Supplemental Table S15). However, in the presence of cladribine alone or in combination with deoxycytidine, microglial cells increased the level of ROS produced (Figure 3j, Supplemental Table S16).

**Figure 4:**
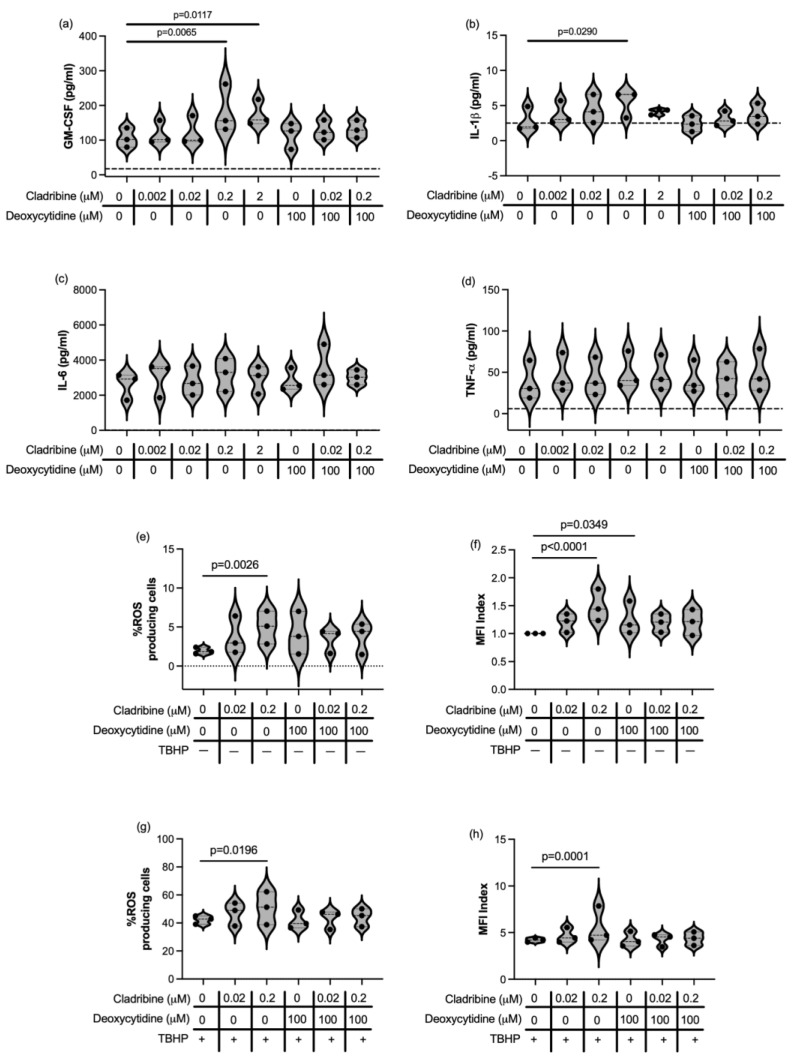
Effect of cladribine in ROS production and in the secretion of GM-CSF, IL-1β, IL-6, and TNF-α in cytokine-stimulated primary human astrocytes. The effect of cladribine on immune function of primary human astrocytes was assessed upon cytokine stimulation (with IL-1β and TNF-α). Release of GM-CSF (**a**), IL-1β (**b**), IL-6 (**c**) and TNF-α (**d**) were assessed by Procartaplex Immunoassay in a DCK dependent manner and independently of DCK activity. The dashed lines indicate the lower limit of quantification for each analyte. Percentage of viable cells positive for CellROX (ROS-producing cells), measured by flow cytometry, respect to basal (**e**,**g**) and CellROX mean fluorescence intensity (MFI) in the total viable population (**f**,**h**) in resting human astrocytes (**e**,**f**) and TBHP-induced (**g**,**h**) human astrocytes are represented. Three independent experiments were performed and each condition was determined in two replicates.

We then assessed the effect of cladribine in the immune function of human astrocytes. We induced the production of granulocyte-macrophage colony-stimulating factor (GM-CSF), IL-6, TNF-α and IL-1β in primary human astrocytes stimulated with cytokines. High concentrations (2 and 0.2 μM) of cladribine enhanced GM-CSF production and 0.2 μM of cladribine increased IL-1β production on cytokine-stimulated primary human astrocytes (Figure 4a,b, Supplemental Tables S17 and S18). In addition, when deoxycytidine was added to the culture to block the DCK pathway in the presence of cladribine, astrocytes presented a cytokine-release pattern similar to that of stimulated astrocytes, effectively restoring cladribine’s effect to basal levels (Figure 4a,b, Supplemental Tables S17 and S18). Importantly, the mean estimated brain exposure of cladribine did not affect astrocyte immune function, and cladribine did not enhance the production of IL-6 or TNF-α (Figure 4c,d, Supplemental Tables S19 and S20).

Astrocytes become activated during inflammation and produce ROS as part of their immune defense mechanisms. However, excessive and sustained ROS production by activated astrocytes can exacerbate neuroinflammation and further contribute to neuronal injury. In view of this, we assessed how cladribine modulates ROS production in astrocytes. In resting primary human astrocytes, only the highest tested dose of cladribine (0.2 μM) induced oxidative-stress, as measured by the percentage of cells producing ROS (Figure 4e, Supplemental Table S21), and the level of ROS production was also affected by deoxycytidine alone (Figure 4f, Supplemental Table S22). However, no detrimental effect was observed when primary human astrocytes were exposed to the maximum mean estimated concentration of cladribine (0.02 μM) that reaches the CNS in MS patients (Figure 4e,f, Supplemental Tables S21 and S22). Consistently, the percentage of astrocytes producing ROS and the level of ROS significantly increased when astrocytes challenged with TBHP were exposed to a high concentration of cladribine (0.2 μM) (Figure 4g,h, Supplemental Tables S23 and S24). Saturation of the DCK pathway decreased the level of ROS production in resting and TBHP-induced human astrocytes, cultured with 0.2 μM of cladribine, thus reverting the effect of the drug (Figure 4e–h).

Altogether, these results indicate that the concentration of cladribine reaching the CNS in MS patients does not adversely impact glial cells and does not modulate the local immune response. These findings suggest a limited direct influence of cladribine on both glial function and immune regulation within the CNS.

### 2.2. Effect of Cladribine on Neural and Oligodendroglial Cells

#### 2.2.1. Cladribine Does Not Impair the Differentiation of Neuronal Progenitors into Neurons

**Figure 5:**
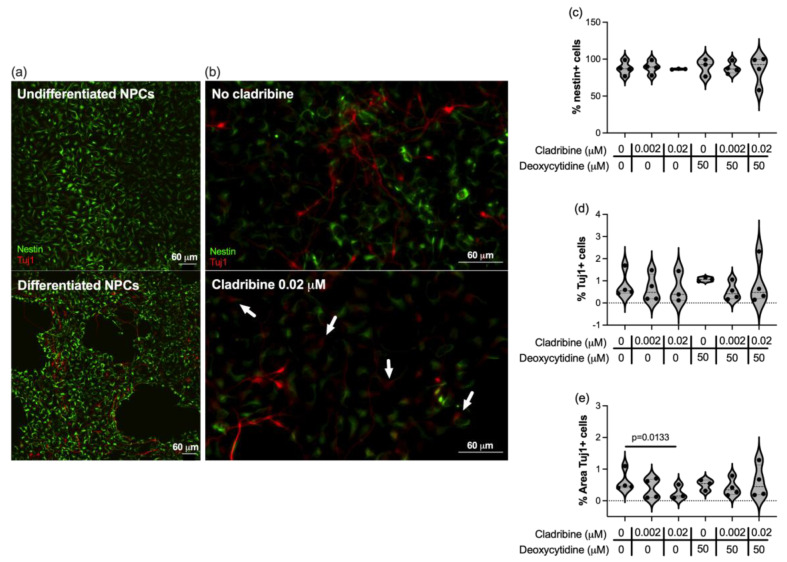
Effect of cladribine in the differentiation of neuronal precursor cells into neurons. Human neuronal precursor cells (ENStem-A Neural progenitor cells) were differentiated for 8 days to lineage-committed immature neurons. The maximum mean estimated brain exposure of cladribine and a lower concentration of the drug (0.02 μM and 0.002 μM) were added to the culture at the time of differentiation, and were maintained until the end of the differentiation period with a 48h wash out period after the first 3 days of differentiation. The differentiation state of neuronal progenitor cells was assessed by immunocytochemistry and the percentage of cells stained with nestin or Tuj1, as well as the area of Tuj1+ cells, were quantified. (**a**) Representative images of undifferentiated and differentiated NPC cultures after 8 days of differentiation. (**b**) Representative images of differentiated NPC cultures in the absence and in the presence of 0.02 μM cladribine. The percentage of nestin-positive cells (**c**), the percentage of Tuj1-positive cells (**d**) and the percentage of Tuj1 area (**e**) were calculated respect to the total number of cells in each well. Nestin is expressed in neuronal progenitors and is represented in green, while Tuj1 is expressed early in differentiating neurons and is represented in red. Arrows in (**b**) indicate neurons in differentiation state with short processes. Four independent experiments were performed and each condition was determined in two replicates.

We assessed how cladribine impacts on the differentiation of neural progenitor cells (NPCs) in the initial stage of the process. To this end, ENStem-A Neural progenitor cells derived from human embryonic stem cells were differentiated for 8 days and exposed to concentrations of cladribine reflecting the mean estimated brain exposure of MS patients (0.02 μM as the maximum concentration reached in the CNS) from the beginning of the differentiation period. After 8 days of differentiation, we observed that NPCs began to differentiate, acquiring the neuronal lineage marker Tuj1 (β III tubulin) and changing their morphology (Figure 5a). The percentage of NPCs expressing nestin (a marker for undifferentiated NPCs) and Tuj1 (a marker for differentiating neurons) did not differ when NPCs were differentiated in the presence of concentrations of cladribine that can reach the CNS (Figure 5c,d, Supplemental Tables S25 and S26). However, the percentage of area occupied by Tuj1 staining was lower when NPCs were cultured in the presence of cladribine at the maximum mean estimated brain exposure (0.02 μM) (Figure 5e, Supplemental Table S27), indicating that neuronal processes in that condition were less represented and were less complex (representative images are shown in Figure 5b). When the DCK activity was blocked by deoxycytidine, the area of Tuj1 staining was comparable to that of differentiated NPCs (basal condition) (Figure 5e, Supplemental Table S27).

#### 2.2.2. Cladribine Does Not Affect the Differentiation of MO3.13 Human Oligodendroglia Cell Line

**Figure 6:**
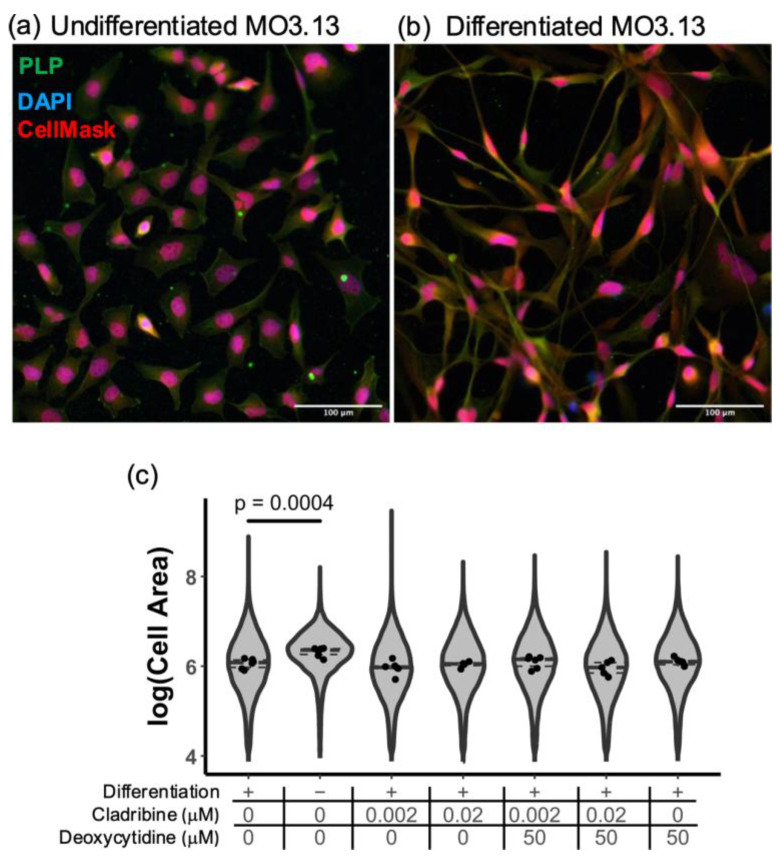
Cladribine does not affect MO3.13 glial cell differentiation. The differentiation state of MO3.13 oligodendroglial cells was determined by immunocytochemistry and the cell area (morphological parameter) was quantified. (**a**) Representative images of undifferentiated and (**b**) differentiated oligodendrocyte-like MO3.13 cells for a period of six days. (**c**) Cladribine does not hinder the capacity of oligodendroglial cells to differentiate. Violins plots in graph (**c**) represent the total number of cells quantified for each condition, and scatter plot represents the mean values for each condition (two replicates for condition). Three independent experiments were performed.

Given the relevance of oligodendrocyte lineage cells in remyelination, we next investigated the potential role of cladribine in modulating oligodendrocyte differentiation using MO3.13 glial cell line, an in vitro model relevant to MS. MO3.13 cells were differentiated for a period of six days. During differentiation, MO3.13 cells undergo marked morphological changes consistent with the progression toward a mature oligodendrocyte-like phenotype [31]. In the undifferentiated state, these cells display a rounded or slightly elongated shape with limited cytoplasmic extensions and low PLP expression (Figure 6a). Upon induction of differentiation, they progressively develop a more complex and branched morphology, and increase the expression of PLP (Figure 6b). These morphological transformations are indicative of a shift toward a differentiated state. Cladribine was added at the initiation of MO3.13 cell differentiation, at concentrations reflecting the mean estimated brain levels that reach the CNS, either alone or in combination with deoxycytidine, and morphological changes were evaluated by immunofluorescence. Undifferentiated MO3.13 cells presented a higher cell area due to their rounded shape, that was significantly reduced upon cell differentiation (Figure 6c, Supplemental Table S28). MO3.13 cells differentiated in the presence of phosphorylated cladribine and non-phosphorylated cladribine showed similar morphology as MO3.13 differentiated cells, suggesting that cladribine does not interfere in the early stages of differentiation process of immature to mature oligodendrocyte-like cells (Figure 6c).

#### 2.2.3. Effect of Cladribine at Estimated Brain Concentrations in Neural- and Oligodendroglial-like Cells Exposed to Inflammatory, Oxidative and Excitotoxic Stress

To investigate the potential neuroprotective effects of cladribine, we employed two differentiated human cell lines with neuronal and oligodendroglial characteristics: SH-SY5Y and MO3.13. Cells were exposed to various stressors that mimic pathological mechanisms in MS, including inflammatory stress induced by IFN-γ and TNF-α, oxidative stress induced by hydrogen peroxide (H_2_O_2_) or TBHP, and, in the case of SH-SY5Y cells, excitotoxic stress induced by glutamate. We then assessed whether cladribine, applied at therapeutically relevant concentrations, could exert protective effects against these cellular insults.

**Figure 7:**
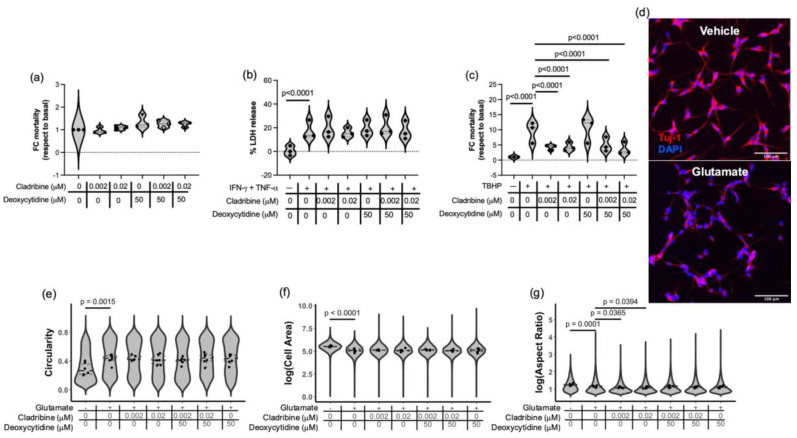
Protective role of steady-state cladribine concentration in neuron-like cells exposed to oxidative insult. SH-SY5Y were differentiated for three days with retinoic acid. Differentiated SH-SY5Y neuron-like cell line were exposed to the maximum mean estimated brain exposure of cladribine and a lower concentration of the drug (0.02 μM and 0.002 μM) at the time that were stimulated with an inflammatory, oxidative and excitotoxic stimulus. (**a**) The concentrations of cladribine used did not increase cell death of differentiated neuron-like cells, which was assessed by flow cytometry. (**b**) Steady-state concentrations of cladribine did not improve the toxicity, measured by release of LDH, related to inflammatory stimulus (exposure to IFN-γ and TNF-α), but (**c**) protected differentiated neuron-like cells from oxidative stress induced with TBHP. (**d**) Morphological changes due to glutamate-induced cytotoxicity in differentiated SH-SY5Y cells were determined by immunocytochemistry. Micrographs showing morphological changes in differentiated SH-SY5Y neuron-like cells (vehicle condition, upper panel) induced by glutamate exposure (lower panel). No protective effect was observed when neuron-like cells were exposed to excitotoxic stimulus (with glutamate) in terms of circularity (loss of pyramidal shape) (**e**), Tuj1 area per cell or cell area (loss of soma volume) (**f**) and aspect ratio (shortened cell length) (**g**). Violins plots in graphs e-g represent the total number of cells quantified for each condition, and scatter plot represent the mean values for each condition (two replicates for condition). Three independent experiments were performed.

SH-SY5Y cells were first differentiated for three days with retinoic acid, resulting in a neuronal phenotype characterized by the presence of branching processes. Differentiated cells were then treated with cladribine at concentrations reflecting the estimated mean maximum levels reached in the brain of MS patients (0.02 μM and 0.002 μM), applied either in its active (phosphorylated) form or combined with deoxycytidine to assess the effect of the non-phosphorylated prodrug. At the tested concentrations, cladribine did not induce cytotoxicity in differentiated SH-SY5Y cells, as shown in Figure 7a and in Supplemental Table S29. However, it did not confer protection against inflammatory stress, as exposure to IFN-γ and TNF-α still resulted in significant cell damage, evidenced by increased lactate dehydrogenase (LDH) release (Figure 7b, Supplemental Table S30), suggesting that under these conditions, cladribine does not exert a protective effect on neuronal-like cells. In contrast, partial protection was observed against toxicity induced by TBHP, a compound associated with oxidative stress. Cell death measured by flow cytometry determined that exposure to TBHP induce mortality in differentiated SH-SY5Y cells by 9.49 ± 3.20 times more (fold-change–FC) respect to basal mortality. In the presence of cladribine alone, 54.33% and 55.50% reduction in FC mortality was observed (0.02 μM and 0.002 μM concentrations, respectively) (Figure 7c, Supplemental Table S31). In addition, this effect was also observed when cladribine was combined with deoxycytydine, reducing FC mortality by 61.51% and 47.33% (deoxycytydine combined with 0.02 μM and 0.002 μM concentrations, respectively) (Figure 7c, Supplemental Table S31), thus acting in a DCK-independent fashion. Notwithstanding, deoxycytidine alone did not protect neural-like cells from oxidative stress (Figure 7c, Supplemental Table S31). These results suggest that cladribine exerts a neuroprotective effect by reducing oxidative-stress mortality.

To model excitotoxic damage, differentiated neuronal-like SH-SY5Y cells were exposed to glutamate in the presence or absence of cladribine. Glutamate treatment induced marked morphological alterations, including neurite retraction and fragmentation, a shift to a rounded morphology, and a significant reduction in soma size, consistent with a degenerative phenotype (Figure 7d). These changes were quantified using morphometric parameters such as increased circularity (Figure 7e, Supplemental Table S32), decreased cell area (Figure 7f, Supplemental Table S33), and reduced aspect ratio (a dimensionless measure of cell elongation, Figure 7g, Supplemental Table S34). Treatment with cladribine, either alone or in combination with deoxycytidine, at the tested concentrations, did not prevent these glutamate-induced alterations. Moreover, a further decrease in aspect ratio was observed in the presence of cladribine alone compared to glutamate treatment alone (Figure 7g, Supplemental Table S34), suggesting a possible interaction between cladribine and excitotoxic mechanisms under these experimental conditions.

**Figure 8:**
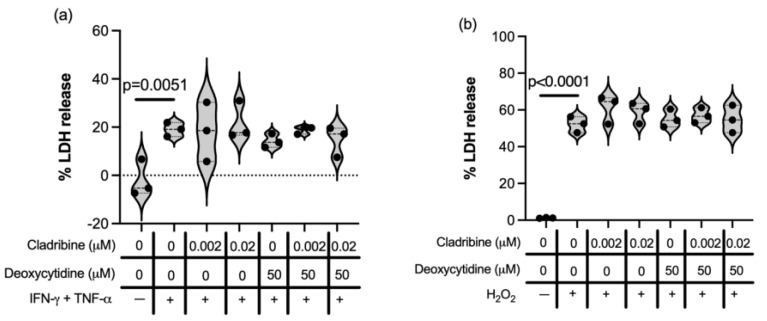
Effect of steady-state cladribine concentration in differentiated MO3.13 oligodendrocyte-like cells exposed to inflammatory and oxidative insult measured by release of LDH. MO3.13 oligodendrocyte-like cell line was differentiated for six days with PMA. Differentiated MO3.13 oligodendrocyte-like cell line was exposed to the maximum mean estimated brain exposure of cladribine and a lower concentration of the drug (0.02 μM and 0.002 μM) at the time that were stimulated with (**a**) an inflammatory insult (48 h exposure with IFN-γ and TNF-α) or with (**b**) an oxidative stress insult (18 h exposure to H_2_O_2_). In both cases, cladribine did not show a protective or deleterious effect in its active or prodrug form. Data represent three independent experiments with two replicates per condition.

In parallel, MO3.13 oligodendrocyte-like cells, previously differentiated with phorbol 12-myristate 13-acetate (PMA) for six days, were subjected to inflammatory stress (induced by IFN-γ and TNF-α; Figure 8a) or oxidative stress (via H_2_O_2_; Figure 8b) in the presence of cladribine at therapeutically relevant concentrations. Under these conditions, cladribine did not exert either protective or deleterious effects, irrespective of whether it was used in its prodrug (DCK-independent pathway) or active (DCK-dependent pathway) form. These findings suggest that, at least in this in vitro oligodendroglial model, cladribine does not directly modulate the cellular response to inflammatory (Supplemental Table S35) or oxidative insults (Supplemental Table S36) and does not enhance the damage produced by inflammation or oxidative stress.

Together, these findings indicate that cladribine, at concentrations corresponding to mean brain levels in MS patients, does not exert general protective effects in oligodendroglial or neural-like cells under inflammatory, oxidative, or excitotoxic stress. Nonetheless, its lack of cytotoxicity—even in the presence of damaging stimuli—supports a favorable cellular safety profile, and partial protection under oxidative conditions warrants further investigation.

Overall, our findings indicate that cladribine, at therapeutically relevant concentrations, does not compromise the viability of CNS resident cells. While it does not protect against inflammatory or excitotoxic insults, it may exert a protective effect against oxidative stress-induced cytotoxicity in neuronal-like cells, through both DCK-dependent and DCK-independent mechanisms. Moreover, cladribine does not impair glial differentiation, although it may influence neurogenic processes. These results suggest that, beyond its well-established immunomodulatory role, cladribine could have context-dependent, non-immune actions within the CNS that may be relevant to MS pathophysiology.

## 3. Discussion

The use of cladribine tablets for treating MS was approved in 2017 by the EMA and in 2019 by the FDA. It is a high efficacy DMT classified as an immune reconstitution therapy (IRT) that selectively and transiently depletes T and B lymphocytes while preserving innate immune function and allowing immune reconstitution, leading to a long-lasting suppression of inflammatory disease activity and long-term clinical benefit. Given that cladribine can cross the BBB, it is reasonable to speculate that the drug may affect CNS-resident cells including neuronal and glial cells. We first investigated the potential effects of cladribine on microglial cells and astrocytes using the human HMC3 cell line and human primary astrocytes. At maximum therapeutic concentrations, cladribine did not alter the viability or proliferative capacity of human astrocytes or microglial cells. These findings are consistent with results from primary microglia cultures derived from mice [32]. However, other studies report that cladribine reduces the proliferative capacity and induces apoptosis of rat microglial cells [33]. The cytotoxic effect of cladribine is directly linked to the accumulation of phosphorylated cladribine, that in turn depends on the DCK/5-NT ratio. Although both these studies used primary cultures, it is important to note that the concentrations of cladribine required to observe effects in murine cells had to be scaled approximately 10-fold to account for the lower ratio of expression of DCK and 5-NT in rodents [34]. In contrast, our in vitro model uses human microglial cells, which more accurately reflect the human DCK/5-NT ratio that allow us to use concentrations of cladribine reflecting the maximum estimated that reaches the CNS, making the findings described here more directly translatable to the clinical setting. However, cladribine did not impair the capacity of microglial cells to produce cytokines at any of the concentrations tested, meaning that cladribine does not have an immunomodulatory effect on human microglial cells. This contrasts with its capacity to modulate the production of pro-inflammatory cytokines by human and murine dendritic cells generated in vitro [34], suggesting an immunomodulatory role of the drug in phagocytic immune cells of the periphery but not in the CNS. Conversely, in human microglial and astrocytes, the production of ROS was enhanced in the presence of cladribine principally in its phosphorylated form. Although excessive or sustained ROS production can lead to oxidative stress and damage to surrounding neurons and other brain cells and contribute to neuroinflammation, physiological or transient production of ROS also plays important signaling roles in both microglia and astrocytes. In microglia, ROS generated by NADPH oxidase act as second messengers in intracellular pathways such as NF-κB and MAPKs, regulating immune responses, promoting controlled phagocytosis, and contributing to synaptic pruning during development and plasticity [35,36]. In astrocytes, mitochondrial ROS participates in the regulation of brain metabolism, thereby influencing neuronal physiology and behavior [37]. Astrocytes also maintain CNS redox balance by producing antioxidants and activating Nrf2-dependent cytoprotective programs, ensuring protection against oxidative stress while supporting normal neuronal function [38]. Due to the short course administration, the maximum therapeutic concentration of cladribine is not maintained for a long period of time [28], suggesting that ROS elevation may be a transient rather than a sustained phenomenon and that it could contribute to the normal functioning of the CNS rather than promoting neuroinflammation and neuronal damage. In fact, these beneficial effects of ROS production could be in line with the observation that cladribine tablets reduced neuroaxonal damage in RRMS [21]. Notwithstanding, cladribine does not dampen neuronal survival as demonstrated in vitro by us and others [39]. However, we observed a reduction in Tuj1-positive area in early stages of NPC differentiation that suggests that cladribine may transiently inhibit or delay neuronal differentiation. Given that peak plasma concentrations are reached within 0.5–1.5 h after oral dosing and decline rapidly, with an elimination half-life of ~1 day [20], sustained exposure at maximum levels is unlikely in vivo. This transient nature and the fact that NPCs were cultured with cladribine for six days, together with the absence of a significant effect once concentrations decrease, suggest that the phenomenon may have limited clinical relevance. These findings reinforce the view that cladribine’s primary impact in the CNS is on immune modulation rather than direct, lasting interference with neuroregeneration [22]. In addition, cladribine that crosses the BBB may dampen the inflammatory activity of infiltrating T and B lymphocytes in focal lesions via direct cytotoxicity, probably lowering pro-inflammatory cytokines and consequently promoting a more permissive environment for neuroregeneration. Its potential neuroprotective effect is supported by the observed reduction in neurofilament light chain levels in MS patients receiving cladribine tablets treatment [40] and by the capacity of oral cladribine treatment to normalize the hyperactivity of the cortical neuronal network induced in the EAE mouse model [41].

In line with this evidence, we found that cladribine protects differentiated SH-SY5Y neuron-like cells from oxidative stress at all tested doses when DCK could phosphorylate the drug; however, this effect also occurs independently of DCK activity. TBHP is well known to induce ROS production and oxidative damage by disrupting cellular antioxidant defenses, among other mechanisms, in different cell types [42,43,44]. Consistently, clinical studies in secondary progressive MS patients have shown that treatment with cladribine tablets improves systemic redox status by lowering serum homocysteine levels and increasing total antioxidant capacity [45], supporting a direct protective role of the active form of cladribine under oxidative stress conditions in neurons. Such a protective mechanism could complement cladribine’s immunomodulatory actions by directly preserving neuronal integrity in neuroinflammatory conditions; however, further investigations are warranted to elucidate this potential protective role. Alternatively, and purely as a speculative hypothesis, it could be considered that cladribine might also exert its protective effect through interaction with adenosine receptors. Previous studies have shown that the non-phosphorylated form of cladribine can bind to adenosine receptors, whereas phosphorylation prevents such binding [23]. Cladribine can interact with both A1 and A2a adenosine receptors, with low affinity to the last one, which are expressed in SH-SY5Y cells [24]. A1 receptor activation is primarily associated with the inhibition of excitotoxic mediators release, that are massively secreted during CNS injury and contribute to neurodegeneration. Conversely, activation of A2a receptors in neurons enhances brain-derived neurotrophic factor (BDNF) signaling and release [46]. The observed protection against TBHP-induced cell death may therefore involve cladribine’s binding to adenosine receptors and subsequent phosphorylation of Erk1/2, which is potentiated in SH-SY5Y cells via both A1 and A2a receptors [24] and Erk1/2 pathway has been previously shown to protect these cells from oxidative stress [47]. Nonetheless, the binding of cladribine to adenosine receptors was not the focus of this study and experiments designed to specifically rule out the protective role of cladribine through this signaling pathway using equivalent concentrations of the drug to those found in the CNS of MS patients treated with cladribine tablets are needed to further clarify this point.

Nonetheless, our study has limitations that should be acknowledged. The experiments were conducted in isolated cell lines or primary cultures, which do not fully recapitulate the complex cellular interactions that occur within the CNS microenvironment. Although cell lines such as SH-SY5Y and MO3.13 offer practical advantages and are of human origin, they are less physiologically representative than primary CNS cultures and do not fully represent the physiological maturation and differentiation status of CNS cell types, although standard culture and differentiation protocols described in the literature were applied. However, the use of human-derived lines avoids species-specific differences inherent to rodent primary cultures, while the alternative—human iPSC-derived differentiated cells—also relies on artificial differentiation protocols and remains limited by variability and maturation state. Thus, while these models have intrinsic constraints, they provide a relevant and tractable system for exploratory studies on the potential immune and non-immune effects of cladribine in the CNS through DCK or independently of DCK activity. To address these limitations, future studies incorporating co-culture systems, organoids, or brain-on-chip platforms could provide a more physiologically relevant context to assess the role of cladribine under pathological conditions resembling MS. In addition, comparative analyses using human iPSC-derived neurons and glial cells may help clarify whether the observed effects are consistent across cell sources and differentiation states, and whether cladribine’s impact is modulated by developmental or metabolic maturity. In addition, reproducing cladribine pharmacokinetics in vitro is challenging, as peak plasma levels occur within 0.5–1.5 h after oral dosing and decline rapidly. This results in transient exposure followed by decreasing concentrations over the following 24 h approximately, while CNS pharmacokinetics remain unknown. To ensure sufficient exposure, cladribine was maintained in culture for extended periods (24–72 h), particularly in differentiation protocols (up to six days), to assess early-stage effects. Although a pulse–washout approach could better mimic in vivo kinetics, our design prioritized continuous exposure to capture potential impacts on differentiation.

To summarize, we described in different human cell models that cladribine, at therapeutic concentrations, does not alter the survival, proliferative and cytokine release capacity of microglial cells and astrocytes. However, it increases the production of reactive oxygen species in both cell types, which could contribute to neuroinflammation but could also participate in neuroprotection due to the transient exposure of glial cells to therapeutic concentrations of cladribine in MS patients. We also described that the tri-phosphorylated form of cladribine partially impairs the initial stages of differentiation process of NPCs but does not influence oligodendroglial differentiation. In terms of direct neuroprotection, cladribine enhanced survival of oxidative-stress-challenged SH-SY5Y neural cells.

## 4. Materials and Methods

### 4.1. Human Primary Cells and Cell Lines, Media and Differentiation Protocols

HMC3 (embryonic microglia clone 3; cat# CRL-3304) and SH-SY5Y (cat# CRL-2266) cell lines were obtained from ATCC (Manassas, VA, USA). Primary human astrocytes isolated from cerebral cortex (cat# 1800) were purchased at ScienCell Research Laboratories (Carlsbad, CA, USA). Primary ENStem-A Human Neural Progenitor cells (cat# SCC003) and MO3.13 cell line (cat# CLU301) were purchased from EMD Millipore (Billerica, MA, USA) and Cederlane (Burlington, ON, Canada), respectively.

HMC3 were cultured in EMEM medium (ATCC) containing 10% fetal bovine serum (FBS, ATCC) and 0.5% penicillin/streptomycin (Biowest, Nuaille, France).

Primary human astrocytes were cultured in T75 flasks (Nunclone, Thermo Fisher Scientific, Waltham, MA, USA) previously coated with 2 μg/cm^2^ of poly-L-lysine (PLL; Innoprot, Derio, Spain) in astrocyte medium (ScienCell Research Laboratories) containing basal medium, 1% astrocyte growth supplement and 1% P/S.

A vial of ENStem-A Human NPCs was thawed and plated in a 35 mm dish (TPP, Trasadingen, Switzerland) previously coated with 20 μg/mL poly-L-ornithine (Sigma, Burlington, MA, USA) and subsequent coated with 5 μg/mL laminin (Merck KGaA, Darmstadt, Germany), and cultured in ENStem-A Neural Expansion Medium (EMD Millipore) supplemented with 20 ng/mL of basic fibroblast growth factor 2 (bFGF-2) and 2 mM L-Glutamine (Biowest). NPC cultures that reached 90–100% confluence were detached via enzymatic dissociation with Accutase (EMD Millipore), and the content of a 35 mm well was seeded in 8 wells of a poly-L-ornithine and laminin-coated 12-well plate for differentiation. ENStem-A Neuronal Differentiation Medium supplemented with 2 mM L-Glutamine was added to NPC cultures reaching 40–50% confluence. Medium was changed every two or three days and, when confluence was reached (typically after three days of differentiation), differentiating NPCs were subcultured into Micro Plate 24-well black–IbiTreat (Ibidi GmbH, Gräfelfing, Germany) coated with poly-L-ornithine and laminin (200,000 cells per well in complete ENStem-A Neuronal Differentiation Medium). NPCs were differentiated for a total of eight days.

SH-SY5Y neuroblastoma cell line was cultured in EMEM (ATCC) and Ham’s F12 (Gibco, Waltham, MA, USA) (1:1) supplemented with 10% FBS and 0.5% P/S in flasks coated with 50 µg/mL collagen IV (Sigma). For differentiation into neural-like cells, depicted wells were coated with 50 µg/mL collagen IV and cells were seeded at 10,000 cells/cm^2^ unless otherwise stated. SH-SY5Y cells were differentiated with complete EMEM/F12 containing 10 µM retinoic acid for a period of three to six days. Differentiating medium was changed every two or three days.

MO3.13 cell line was cultured in DMEM medium (Gibco) supplemented with 10% FBS and 0.5% P/S. For differentiation experiments, 15,000 cells were seeded in 24-well plates with coverslips coated with 100 μg/mL poly-D-lysine (Sigma). After 48 h, when cultures reached 70% of confluence, wells were washed with non-supplemented DMEM, and differentiation medium (DMEM without FBS and supplemented with 100 nM PMA, from Sigma) was added and was replaced every two days [31]. MO3.13 cells were differentiated for six days. In experiments where coverslips were not used, no coating was required.

### 4.2. Cladribine and Deoxycytidine

Cladribine (Merck KGaA) was dissolved in dimethyl sulfoxide (DMSO) at 10 mM, and further diluted to the appropriate concentration in complete medium. Concentrations of cladribine were chose taking into account that the 25% of cladribine levels that are found in blood reach the CNS (ranging from 0.0175 µM to 0.025 µM) [20]. We used the 0.02 µM concentration as the mean brain concentration of cladribine, scale up to 0.2 and 2 µM and scale down to a lower concentration (0.002 µM). Deoxycytidine (Sigma) was dissolved in water and further diluted to the appropriate concentration in complete medium. Deoxycytidine was used at excess amounts (ranging from 25,000-fold to 50-fold molar excess) [48], and 100 μM (HMC3 and human astrocytes) or 50 μM (NPCs, SH-SY5Y and MO3.13 cells) were used. When used in combination with cladribine, deoxycytidine was pre-incubated with the cells for 30 min prior to the addition of cladribine. In all experiments, equivalent volumes of DMSO and water were added to non-treated cultures.

In differentiation experiments, cladribine and/or deoxycytidine were added at the beginning of the differentiation period (NPCs and MO3.13). In the case of NPCs, when cells were split into Micro Plate 24-well black–IbiTreat, they were cultured in differentiation medium for 48 h (wash out period) to either facilitate their recovery and adherence to the plate, and then cladribine and/or deoxycytidine were added to the appropriate concentrations for additional 72 h.

### 4.3. Cytokine Stimulation

HMC3 were seeded in 6-well plates (90,000 cells/well) and stimulated for 27 h with 10 ng/mL IL-1β (Peprotech, Waltham, MA, USA), 100 ng/mL IFN-γ (Sigma) and 100 ng/mL TNF-α (Peprotech) in the presence of cladribine and/or deoxycytidine at the indicated concentrations. Then, medium was withdrawn, wells were washed twice with PBS1x and fresh complete EMEM medium was added to all wells containing cladribine and/or deoxycytidine. Cells were cultured for additional 44 h, supernatants were harvested and properly stored at −80 °C for cytokine release assay, and activation status was assessed by flow cytometry. Briefly, the HMC3 cells were stained with FITC anti-human HLA-DR antibody (clone L243, Biolegend, San Diego, CA, USA) after blocking Fc receptors with FcBlock (Becton Dickinson, Drive Franklin Lakes, NJ, USA). Cells were acquired with a CytoFlex cytometer (Beckman Coulter, Brea, CA, USA) and analyzed with Cytexpert software 2.0 (Beckman Coulter).

Human astrocytes were seeded in 24-well plates (40,000 cells/well) and stimulated for 6 h with 20 ng/mL human IL-1β + 20 ng/mL human TNF-α in the presence of cladribine and/or deoxycytidine. Then, stimulus was withdrawn and cells were washed trice with non-complete astrocyte medium, and fresh complete astrocyte medium containing cladribine and/or deoxycytidine was added to dedicated wells. Cells were left in culture for additional 18 h, then supernatants were harvested and stored at −80 °C to quantify cytokine release with Procartaplex Immunoassay (see Section 4.6).

For cytokine insult experiments, 40,000 SH-SY5Y and 10,000 MO3.13 cells were initially seeded in 96-flat bottom-well plates and differentiated for three and four days, respectively. Wells were precoated with collagen IV in the case of SH-SY5Y cells. After that period, cells were stimulated with 100 ng/mL IFN-γ and 100 ng/mL TNF-α in the presence of cladribine and/or deoxycytidine for additional 72 h (SH-SY5Y cells) and 48 h (MO3.13 cells) in differentiation medium. Then, cell toxicity was assessed lactate dehydrogenase (LDH) release (see Section 4.8).

### 4.4. Determination of Cell Death

Cell death was assayed in resting HMC3 cell line, resting human astrocytes and three-days differentiated SH-SY5Y cells by flow cytometry with Fixable Viability Dye eFluor 450 (Thermo Fisher Scientific), after 72 h culture in the presence of cladribine and/or deoxycytidine, with differentiation medium in the case of SH-SY5Y cells. Cells were acquired with a CytoFlex cytometer (Beckman Coulter) and analyzed with Cytexpert software 2.0 (Beckman Coulter).

### 4.5. Proliferation Assay

For proliferation assays in HMC3 cells and human astrocytes, Click-iT^®^ Plus EdU flow cytometry assay (Thermo Fisher Scientific) was used. Specifically, 150,000 HMC3 cells were seeded in 6-well plates, and 20,000 human astrocytes were plated in coated 24-well dishes, and were cultured for 72 h with cladribine and/or deoxycytidine. EdU (5-ethynyl-2′-deoxyuridine) was added at the end of the proliferation period at a final concentration of 10 mM for 3 h or 4 h, respectively. Cells were detached and staining for proliferation determination was performed following manufacturer’s instructions. Cells were acquired with a CytoFlex cytometer (Beckman Coulter) and analyzed with Cytexpert software 2.0 (Beckman Coulter).

### 4.6. Cytokine Release Quantification

IL-6, IL-1β and TNF-α were quantified in the supernatants of cytokine-stimulated HMC3 cells, and IL-6, IL-1β, TNF-α and GM-CSF were quantified in the supernatants of cytokine-stimulated human astrocytes using ProcartaPlex Custom Human Immunoassay (Thermo Fisher Scientific), according to the manufacturer’s instructions. Samples were acquired in a Luminex Magpix instrument (Thermo Fisher Scientific) and data were analyzed with ProcartaPlex Analyst 1.0 software (Thermo Fisher Scientific).

### 4.7. Determination of Oxidative Stress

Oxidative stress was determined with CellROX Deep Red flow cytometry assay (Molecular Probes, Thermo Fisher Scientific) in HMC3 and human astrocyte cells. Briefly, 90,000 HMC3 cells and 40,000 human astrocytes were seeded in 6-well and coated 24-well plate, respectively. Cells were cultured for 24 h in the presence of different concentrations of cladribine and/or deoxycytidine. Then, medium was removed and cells were challenged with 400 μM of TBHP for 30 min in normal growth conditions, and CellROX was added at 500 nM for additional 30 min. Cells were detached, washed and stained with SYTOX blue to exclude death cells from the analysis. Cells were acquired immediately in a CytoFlex cytometer (Beckman Coulter) and data was analyzed with Cytexpert software 2.0 (Beckman Coulter).

For SH-SY5Y cell line, 20,000 cells were seeded in collagen IV coated 24-well plates, differentiated for three days and challenged with 800 μM TBHP (Sigma) for 1 h and 20 min. After oxidative stress insult withdrawal, cells were cultured in the presence of cladribine and/or deoxycytidine for additional 48 h in differentiation medium. Cytotoxicity was measured by means of percentage of dead cells assessed by flow cytometry, staining cells with Fixable Viability Dye eFluor 450 (Thermo Fischer Scientific).

For MO3.13 cell line, 10,000 cells were initially seeded in 96-flat bottom-well plates and differentiated for five days. Then, different concentrations of cladribine in combination with deoxycytidine or alone were added to the cultures in differentiation medium, cells were simultaneously challenged with 100 μM H_2_O_2_ (Sigma) and cultures were maintained for 18 h. Cytotoxicity was assessed by LDH release (see Section 4.8).

### 4.8. Cytotoxicity Detection by LDH Release

Cell toxicity was assessed by quantifying LDH activity released from damaged cells using the Cytotoxicity Detection Kit Plus (LDH) from Roche Diagnostics (Mannheim, Germany), following manufacturer’s instructions. To monitor the interference of the drug/substance in LDH determination, a control well containing the maximum concentration of substances used was included. The assay was performed in 96-well plates directly into cells, and samples were run in triplicate. Absorbance was measured at 492 nm and 692 nm (as the reference wavelength). The mean net optical density (OD) (492 nm measure–692 nm measure) was calculated for each sample, and the percentage of LDH release (cytotoxicity) was calculated using the mean net OD for each sample as follows:

$$\%\;LDH\;release=\left(Sample\;net\;OD-Low\;control\;net\;OD\right)/(High\;control\;net\;OD-Low\;control\;net\;OD)$$

### 4.9. Excitotoxic Insult in Differentiated SH-SY5Y Cells

Excitotoxicity was assessed in three-day differentiated SH-SY5Y cells. First, 20,000 undifferentiated SH-SY5Y cells were seeded in collagen IV pre-coated micro-plate 24-well black IbiTreat to induce their differentiation for a period of three days. Glutamate (150 mM, Sigma), cladribine (at different concentrations) and/or deoxycytidine were added to the culture in differentiation medium for 24 h. Cytotoxicity was assessed by morphological parameter determination.

For all stimuli, equivalent volumes of solvents were added to non-stimulated cultures.

### 4.10. Immunocytochemistry

Cells were fixed with 4% paraformaldehyde (PFA) for 15 min. After 3 washes with PBS1x, differentiated NPCs and glutamate-challenged differentiated SH-SY5Y were incubated for 30 min at room temperature (RT) with blocking and permeabilization solution containing 5% normal mouse serum (NMS; Sigma) and 0.5% triton X-100 (Sigma). Primary antibodies were diluted in staining solution containing 1% NMS and 0.1% triton X-100. To assess NPC differentiation status, AF488-conjugated anti-nestin (1:250, Merck KGaA) and AF594-conjugated anti-Tuj1 (1:1000, Biolegend) were used. To assess cytotoxicity, glutamate-challenged differentiated SH-SY5Y cells were stained with AF594-conjugated Tuj1 (1:1000). All primary antibodies were incubated overnight (ON) at 2–4 °C. HCS CellMask Far Red (1:10,000; Thermo Fisher Scientific) was used in differentiated NPCs to easily visualize individual cells regardless the intensity of antigen-specific expression in the different cellular structures. Coverslips were mounted directly inside the wells in Ibidi mounting medium with DAPI (Ibidi GmbH).

Fixed differentiated MO3.13 cells were first permeabilized with 0.1% triton X-100 solution for a total of 15 min at RT, refreshing the permeabilization solution every 5 min. Then, MO3.13 cells were blocked with 5% normal goat serum (NGS; Merck KGaA) for 1 h at RT. Purified anti-PLP (1:100, Merck KGaA) was diluted in staining solution containing 1% NGS and was incubated ON at 2–4 °C. Secondary antibody AF488-conjugated goat anti-mouse H + L (1:500, Thermo Fisher) was incubated for 1 h at RT. HCS CellMask Far Red (1:10,000) was used to easily visualize individual cells regardless the intensity of antigen-specific expression in the different cellular structures. Nucleis were stained with DAPI (1:1000, Sigma) and coverslips were mounted with Fluoromount G mounting medium (Thermo Fisher Scientific) in Superfrost Plus Gold microscope slides (Thermo Fisher Scientific).

### 4.11. Image Acquisition and Analysis

Images were acquired using a Leica AF6000 fluorescence microscope (Leica Microsystems, Wetzlar, Germany), and were obtained at a 20× magnification. A mosaic image of each well was acquired by using the LASX Navigator software (Leica Microsystems), and quantifications were automated using workflows (macro script) in Image J software [49,50] in order to avoid vias, and to be able to analyze a range of 2000 to 10,000 cells per well.

For NPC differentiation experiments, fluorescence tiles were acquired from cells labeled with DAPI, nestin (neural progenitor marker), Tuj1 (neural differentiation marker) and HCS CellMask (discriminate dead cells and segmentation of healthy cells). We quantified the percentage of cells expressing nestin and Tuj1 respect to the total number of cells, and the area of Tuj1 staining respect to the total area of cells.

For MO3.13 differentiation, fluorescence tiles were acquired from cells labeled with DAPI, PLP (oligodendroglia marker) and HCS CellMask. Area of PLP (cell area) and morphological parameters (circularity and aspect ratio) were measured. Aspect ratio is a dimensionless parameter that indicates the degree of length of a cellular structure. 

Cell toxicity in glutamate-challenged SH-SY5Y cells was assessed by measurement of morphological parameters of neuron-like cells stained with Tuj1. Fluorescence tiles were acquired from cells labeled with DAPI and Tuj1. Area of Tuj1 (cell area) and morphological parameters (circularity and aspect ratio) were measured.

### 4.12. Statistical Analysis

Mixed-effects models were used to account for correlated measures, with culture condition as a fixed effect and experiment as a random effect. Within each experiment, the different culture conditions were compared as paired observations, and technical replicates were modeled as repeated measures within each condition.

Depending on the data distribution, either beta (for bounded ratios) or gamma (for positive continuous variables) generalized linear mixed models (GLMMs) were fitted.

Estimated marginal means were obtained, and multiple comparisons were performed with Dunnett’s post hoc test, taking as reference the stimulated condition (for cytokine-, oxidative stress-, or glutamate-challenge experiments) or the basal condition (for survival, proliferation, and differentiation assays), as stated in each figure legend.

## 5. Conclusions

We conclude that, overall, cladribine may exert a protective rather than a detrimental role in the CNS, which is relevant for MS patients receiving this treatment, and that the drug can have effects beyond lymphocyte depletion.

## Data Availability

Data will be made available upon reasonable request to corresponding authors (carmen.espejo@vhir.org; herena.eixarch@vhir.org).

## Supplementary Materials

The following supporting information can be downloaded at: https://www.mdpi.com/article/10.3390/ijms262311311/s1.
