# Supplementary material for: Cladribine Preserves Normal Central Nervous System Cellular Activity and Promotes Neuroprotection to Oxidative Stress Damage
Source: Int J Mol Sci. 2025 Nov 22;26(23):11311. doi: 10.3390/ijms262311311 (PMC12691795; doi:10.3390/ijms262311311)
Supplement: Supplementary file 1 [file ijms-26-11311-s001.zip › ijms-3912366-supplementary.pdf]

Supplemental Table S1 – Effect size and p-value of %Cell Death in HMC3 cells exposed to cladribine (corresponding to Figure 1a). Multiple comparisons were performed with Dunnett's post-hoc test, taking as reference the basal condition.

**Contrast**	**ODDS ratio**	**p-value**
0.002 μM CDB - Basal	0.985	0.9994
0.02 μM CDB - Basal	1.045	0.9923
0.2 μM CDB - Basal	6.339	<0.0001
2 μM CDB - Basal	35.428	<0.0001

CDB: cladribine

Supplemental Table S2 – Effect size and p-value of %proliferation in HMC3 cells exposed to cladribine (corresponding to Figure 1b). Multiple comparisons were performed with Dunnett's post-hoc test, taking as reference the basal condition.

**Contrast**	**ODDS ratio**	**p-value**
0.002 μM CDB - Basal	0.5620	0.3544
0.02 μM CDB - Basal	1.1007	0.9845
0.2 μM CDB - Basal	0.1072	<0.0001
2 μM CDB - Basal	0.0855	<0.0001

CDB: cladribine

Supplemental Table S3 – Effect size and p-value of %Cell Death in HMC3 cells exposed to cladribine in a DCK-dependent and independent manner (corresponding to Figure 1c). Multiple comparisons were performed with Dunnett's post-hoc test, taking as reference the basal condition.

**Contrast**	**ODDS ratio**	**p-value**
0.02 μM CDB - Basal	0.953	0.9601
0.2 μM CDB - Basal	2.457	<0.0001
DEO - Basal	0.849	0.4074
0.02 μM CDB+DEO - Basal	1.239	0.1155
0.2 μM CDB +DEO - Basal	1.262	0.0718

CDB: cladribine; DEO: deoxycytidine

Supplemental Table S4 – Effect size and p-value of %Proliferation in HMC3 cells exposed to cladribine in a DCK-dependent and independent manner (corresponding to Figure 1d). Multiple comparisons were performed with Dunnett's post-hoc test, taking as reference the basal condition.

**Contrast**	**ODDS ratio**	**p-value**
0.02 μM CDB - Basal	1.4482	0.3418
0.2 μM CDB - Basal	0.0633	<0.0001
DEO - Basal	1.3085	0.6686
0.02 μM CDB+DEO - Basal	1.4672	0.3130
0.2 μM CDB +DEO - Basal	1.3975	0.4357

CDB: cladribine; DEO: deoxycytidine

Supplemental Table S5 – Effect size and p-value of %Cell Death in p
rimary human astrocytes exposed to cladribine in a DCK-dependent and independent manner
(corresponding to Figure 2a). Multiple comparisons were performed with Dunnett's post-hoc test, taking as reference the basal condition.

Contrast	Estimate	p-value
0.002 µM CDB - Basal	-0.1240	1.0000
0.02 µM CDB - Basal	0.05107	1.0000
0.2 µM CDB - Basal	4.0079	0.1563
2 µM CDB - Basal	32.7886	<0.0001
DEO - Basal	0.3931	1.0000
0.02 µM CDB+DEO - Basal	0.3234	1.0000
0.2 µM CDB +DEO - Basal	0.7523	0.9990

CDB: cladribine; DEO: deoxycytidine

Supplemental Table S6 – Effect size and p-value of %Proliferation in primary human astrocytes exposed to cladribine in a DCK-dependent and independent manner
(corresponding to Figure 2b). Multiple comparisons were performed with Dunnett's post-hoc test, taking as reference the basal condition.

Contrast	Estimate	p-value
0.002 µM CDB - Basal	0.9124	0.9478
0.02 µM CDB - Basal	-1.2846	0.7924
0.2 µM CDB - Basal	-20.5924	<0.0001
2 µM CDB - Basal	-31.7439	<0.0001
DEO - Basal	0.3529	0.9999
0.02 µM CDB+DEO - Basal	1.7313	0.5978
0.2 µM CDB +DEO - Basal	-0.1068	1.0000

CDB: cladribine; DEO: deoxycytidine

Supplemental Table S7 – Effect size and p-value of %HLA-DR expression on stimulated HMC3 cells exposed to cladribine (corresponding to Figure 3a). Multiple comparisons were performed with Dunnett's post-hoc test, taking as reference the stimulated condition.

Contrast	ODDS ratio	p-value
0.002 µM CDB - Stimulated	1.001	1.0000
0.02 µM CDB - Stimulated	0.975	0.9937
0.2 µM CDB - Stimulated	0.883	0.7707
2 µM CDB - Stimulated	1.050	0.9708

CDB: cladribine

Supplemental Table S8 – Effect size and p-value of IL-1beta (pg/ml) release by stimulated HMC3 cells exposed to cladribine (corresponding to Figure 3b). Multiple comparisons were performed with Dunnett's post-hoc test, taking as reference the stimulated condition.

**Contrast**	**ODDS ratio**	**p-value**
0.002 µM CDB - Stimulated	0.742	0.8181
0.02 µM CDB - Stimulated	0.741	0.8172
0.2 µM CDB - Stimulated	0.613	0.5185
2 µM CDB - Stimulated	1.256	0.9009

CDB: cladribine

Supplemental Table S9 – Effect size and p-value of IL-6 (pg/ml) release by stimulated HMC3 cells exposed to cladribine (corresponding to Figure 3c). Multiple comparisons were performed with Dunnett's post-hoc test, taking as reference the stimulated condition.

**Contrast**	**ODDS ratio**	**p-value**
0.002 µM CDB - Stimulated	1.15	0.6065
0.02 µM CDB - Stimulated	1.25	0.1821
0.2 µM CDB - Stimulated	1.19	0.3625
2 µM CDB - Stimulated	1.38	0.0194

CDB: cladribine

Supplemental Table S10 – Effect size and p-value of TNF-alpha (pg/ml) release by stimulated HMC3 cells exposed to cladribine (corresponding to Figure 3d). Multiple comparisons were performed with Dunnett's post-hoc test, taking as reference the stimulated condition.

**Contrast**	**ODDS ratio**	**p-value**
0.002 µM CDB - Stimulated	0.684	0.0639
0.02 µM CDB - Stimulated	0.778	0.3263
0.2 µM CDB - Stimulated	0.672	0.0439
2 µM CDB - Stimulated	1.059	0.9678

CDB: cladribine

Supplemental Table S11 – Effect size and p-value of %HLA-DR expression in stimulated HMC3 cells exposed to cladribine in a DCK-dependent and independent manner (corresponding to Figure 3e). Multiple comparisons were performed with Dunnett's post-hoc test, taking as reference the stimulated condition.

**Contrast**	**ODDS ratio**	**p-value**
0.02 µM CDB - Stimulated	1.034	0.8029
0.2 µM CDB - Stimulated	0.917	0.1029
DEO - Stimulated	1.045	0.6348
0.02 µM CDB+DEO - Stimulated	1.043	0.6612

0.2 μM CDB +DEO - Stimulated	1.017	0.9673

CDB: cladribine; DEO: deoxycytidine

Supplemental Table S12 – Effect size and p-value of of IL-1beta (pg/ml) release by stimulated HMC3 cells exposed to cladribine in a DCK-dependent and independent manner (corresponding to Figure 3f). Multiple comparisons were performed with Dunnett's post-hoc test, taking as reference the stimulated condition.

**Contrast**	**Estimate**	**p-value**
0.02 μM CDB - Stimulated	-2.29	0.0697
0.2 μM CDB - Stimulated	2.07	0.4167
DEO - Stimulated	-2.29	0.0697
0.02 μM CDB+DEO - Stimulated	3.72	0.0625
0.2 μM CDB +DEO - Stimulated	2.53	0.3071

CDB: cladribine; DEO: deoxycytidine

Supplemental Table S13 – Effect size and p-value of of IL-6 (pg/ml) release by stimulated HMC3 cells exposed to cladribine in a DCK-dependent and independent manner (corresponding to Figure 3g). Multiple comparisons were performed with Dunnett's post-hoc test, taking as reference the stimulated condition.

**Contrast**	**ODDS ratio**	**p-value**
0.02 μM CDB - Stimulated	1.26	0.6864
0.2 μM CDB - Stimulated	1.25	0.7055
DEO - Stimulated	1.04	0.9966
0.02 μM CDB+DEO - Stimulated	1.18	0.8623
0.2 μM CDB +DEO - Stimulated	1.33	0.5221

CDB: cladribine; DEO: deoxycytidine

Supplemental Table S14 – Effect size and p-value of of TNF-alpha (pg/ml) release by stimula ted HMC3 cells exposed to cladribine in a DCK-dependent and independent manner (corresponding to Figure 3h). Multiple comparisons were performed with Dunnett's post-hoc test, taking as reference the stimulated condition.

**Contrast**	**ODDS ratio**	**p-value**
0.02 μM CDB - Stimulated	1.25	0.5894
0.2 μM CDB - Stimulated	1.04	0.9953
DEO - Stimulated	1.16	0.8344
0.02 μM CDB+DEO - Stimulated	1.36	0.3002
0.2 μM CDB +DEO - Stimulated	1.56	0.0610

CDB: cladribine; DEO: deoxycytidine

Supplemental Table S15 – Effect size and p-value of % ROS producing HMC3 cells exposed to cladribine in a DCK-dependent and independent manner (corresponding to Figure 3i). Multiple comparisons were performed with Dunnett's post-hoc test, taking as reference the TBHP-stimulated condition.

**Contrast**	**ODDS ratio**	**p-value**
Basal – TBHP stimulated	0.00288	<0.0001
NAC+TBHP - TBHP stimulated	0.14007	<0.0001
0.02 μM CDB - TBHP stimulated	1.52677	0.2356
0.2 μM CDB - TBHP stimulated	0.57794	0.0124
DEO - TBHP stimulated	1.48900	0.2857
0.02 μM CDB+DEO - TBHP stimulated	1.56983	0.1871
0.2 μM CDB +DEO - TBHP stimulated	1.444464	0.3550

CDB: cladribine; DEO: deoxycytidine; NAC: N-acetylcysteine;
TBHP: tert-butyl hydroperoxide

Supplemental Table S16 – Effect size and p-value of MFI Index in TBHP-stimulated HMC3 cells producing ROS and exposed to cladribine in a DCK-dependent and independent manner (corresponding to Figure 3j). Multiple comparisons were performed with Dunnett's post-hoc test, taking as reference the TBHP-stimula ted condition.

**Contrast**	**Estimate**	**p-value**
NAC+TBHP - TBHP stimulated	-40.7	<0.0001
0.02 μM CDB - TBHP stimulated	27.8	0.0002
0.2 μM CDB - TBHP stimulated	43.0	<0.0001
DEO - TBHP stimulated	30.4	0.0001
0.02 μM CDB+DEO - TBHP stimulated	23.7	0.0015
0.2 μM CDB +DEO - TBHP stimulated	16.8	0.0297

CDB: cladribine; DEO: deoxycytidine; NAC: N-acetylcysteine;
TBHP: tert-butyl hydroperoxide

Supplemental Table S17 – Effect size and p-value of GM-CSF production in cytokine-stimulated primary human astrocytes exposed to cladribine in a DCK-dependent and independent manner (corresponding to Figure 4a). Multiple comparisons were performed with Dunnett's post-hoc test, taking as reference the stimulated condition.

**Contrast**	**Estimate**	**p-value**
0.002 μM CDB - Stimulated	6.4561	0.9997
0.02 μM CDB - Stimulated	13.8247	0.9674
0.2 μM CDB - Stimulated	73.5380	0.0065
2 μM CDB - Stimulated	68.2293	0.0117
DEO - Stimulated	5.8187	0.9999
0.02 μM CDB+DEO - Stimulated	20.2803	0.8223
0.2 μM CDB +DEO - Stimulated	23.5933	0.7090

CDB: cladribine; DEO: deoxycytidine

Supplemental Table S18 – Effect size and p-value of IL-1beta production in cytokine-stimulated primary human astrocytes exposed to cladribine in a DCK-dependent and independent manner (corresponding to Figure 4b). Multiple comparisons were performed with Dunnett's post-hoc test, taking as reference the stimulated condition.

Contrast	Estimate	p-value
0.002 μM CDB - Stimulated	0.4456	0.9883
0.02 μM CDB - Stimulated	1.1415	0.4982
0.2 μM CDB - Stimulated	2.3225	0.0290
2 μM CDB - Stimulated	1.2423	0.4131
DEO - Stimulated	-0.7492	0.8514
0.02 μM CDB+DEO - Stimulated	0.02611	1.0000
0.2 μM CDB +DEO - Stimulated	0.7956	0.8147

CDB: cladribine; DEO: deoxycytidine

Supplemental Table S19 – Effect size and p-value of IL-6 production in cytokine-stimulated primary human astrocytes exposed to cladribine in a DCK-dependent and independent manner (corresponding to Figure 4c). Multiple comparisons were performed with Dunnett's post-hoc test, taking as reference the stimulated condition.

Contrast	Estimate	p-value
0.002 μM CDB - Stimulated	406.46	0.8301
0.02 μM CDB - Stimulated	191.20	0.9966
0.2 μM CDB - Stimulated	604.32	0.4866
2 μM CDB - Stimulated	344.09	0.9130
DEO - Stimulated	228.92	0.9896
0.02 μM CDB+DEO - Stimulated	954.55	0.1097
0.2 μM CDB +DEO - Stimulated	428.55	0.7952

CDB: cladribine; DEO: deoxycytidine

Supplemental Table S20 – Effect size and p-value of TNF-alpha production in cytokine- stimulated primary human astrocytes exposed to cladribine in a DCK-dependent and independent manner (corresponding to Figure 4d). Multiple comparisons were performed with Dunnett's post-hoc test, taking as reference the stimulated condition.

Contrast	Estimate	p-value
0.002 μM CDB - Stimulated	9.0668	0.6676
0.02 μM CDB - Stimulated	5.0406	0.9683
0.2 μM CDB - Stimulated	13.2582	0.2978
2 μM CDB - Stimulated	6.3628	0.9038
DEO - Stimulated	2.9527	0.9988
0.02 μM CDB+DEO - Stimulated	2.8650	0.9990
0.2 μM CDB +DEO - Stimulated	8.5825	0.7156

CDB: cladribine; DEO: deoxycytidine

Supplemental Table S21 – Effect size and p-value of % ROS producing resting primary human astrocytes exposed to cladribine in a DCK-dependent and independent manner (corresponding to Figure 4e). Multiple comparisons were performed with Dunnett's post-hoc test, taking as reference the basal condition.

Contrast	ODDS ratio	p-value
0.02 μM CDB - Basal	1.74	0.1507
0.2 μM CDB – Basal	2.43	0.0026
DEO - Basal	1.89	0.0692
0.02 μM CDB+DEO - Basal	1.71	0.1755
0.2 μM CDB +DEO - Basal	1.84	0.0922

CDB: cladribine; DEO: deoxycytidine

Supplemental Table S22 – Effect size and p-value of MFI Index of resting p rimary human astrocytes producing ROS, exposed to cladribine in a DCK-dependent and independent manner (corresponding to Figure 4f). Multiple comparisons were performed with Dunnett's post-hoc test, taking as reference the basal condition.

Contrast	ODDS ratio	p-value
0.02 μM CDB - Basal	1.20	0.1282
0.2 μM CDB - Basal	1.49	<0.0001
DEO - Basal	1.25	0.0349
0.02 μM CDB+DEO - Basal	1.19	0.1430
0.2 μM CDB +DEO - Basal	1.20	0.1147

CDB: cladribine; DEO: deoxycytidine

Supplemental Table S23 – Effect size and p-value of % ROS producing TBHP-stimulated primary human astrocytes, exposed to cladribine in a DCK-dependent and independent manner (corresponding to Figure 4g). Multiple comparisons were performed with Dunnett's post-hoc test, taking as reference the TBHP-stimulated condition.

Contrast	ODDS ratio	p-value
0.02 μM CDB - TBHP stimulated	1.207	0.3796
0.2 μM CDB - TBHP stimulated	1.407	0.0196
DEO - TBHP stimulated	0.981	0.9978
0.02 μM CDB+DEO - TBHP stimulated	1.032	0.9912
0.2 μM CDB +DEO - TBHP stimulated	1.082	0.9035

CDB: cladribine; DEO: deoxycytidine; TBHP: tert-butyl hydroperoxide

Supplemental Table S24 – Effect size and p-value of MFI Index of TBHP-stimulated primary human astrocytes producing ROS, exposed to cladribine in a DCK-dependent and independent manner (corresponding to Figure 4h). Multiple comparisons were performed with Dunnett's post-hoc test, taking as reference the TBHP-stimulated condition.

Contrast	ODDS ratio	p-value
0.02 μM CDB - TBHP stimulated	1.099	0.3929
0.2 μM CDB - TBHP stimulated	1.301	0.0001
DEO - TBHP stimulated	0.994	0.9995
0.02 μM CDB+DEO - TBHP stimulated	1.007	0.9991
0.2 μM CDB +DEO - TBHP stimulated	1.030	0.9582

CDB: cladribine; DEO: deoxycytidine; TBHP: tert-butyl hydroperoxide

Supplemental Table S25 – Effect size and p-value of % differentiating neural precursor cells (NPC) expressing nestin, exposed to cladribine in a DCK-dependent and independent manner (corresponding to Figure 5c). Multiple comparisons were performed with Dunnett's post-hoc test, taking as reference the basal condition.

Contrast	Estimate	p-value
0.002 μM CDB - Basal	1.3334	0.9989
0.02 μM CDB - Basal	2.3288	0.9909
DEO - Basal	1.4594	0.9991
0.002 μM CDB+DEO - Basal	0.3705	1.0000
0.02 μM CDB +DEO - Basal	-2.8494	0.9737

CDB: cladribine; DEO: deoxycytidine

Supplemental Table S26 – Effect size and p-value of % differentiating neural precursor cells (NPC) expressing Tuj1, exposed to cladribine in a DCK-dependent and independent manner (corresponding to Figure 5d). Multiple comparisons were performed with Dunnett's post-hoc test, taking as reference the basal condition.

Contrast	Estimate	p-value
0.002 μM CDB - Basal	-0.1486	0.9467
0.02 μM CDB - Basal	-0.2161	0.8584
DEO - Basal	0.09634	0.9944
0.002 μM CDB+DEO - Basal	-0.2877	0.6179
0.02 μM CDB +DEO - Basal	0.1198	0.9786

CDB: cladribine; DEO: deoxycytidine

Supplemental Table S27 – Effect size and p-value of % area of Tuj1 of neural p recursor cells (NPC)-differentiating cultures exposed to cladribine in a DCK-dep endent and independent manner (corresponding to Figure 5e). Multiple comparisons were performed with Dunnett's post-hoc test, taking as reference the basal condition.

**Contrast**	**Estimate**	**p-value**
0.002 μM CDB - Basal	-0.2183	0.1991
0.02 μM CDB - Basal	-0.4222	0.0133
DEO - Basal	-0.1806	0.4377
0.002 μM CDB+DEO - Basal	-0.1954	0.2882
0.02 μM CDB +DEO - Basal	-0.02384	0.9996

CDB: cladribine; DEO: deoxycytidine

Supplemental Table S28 – Effect size and p-value of Cell Area of diff erentiating MO3.13 exposed to cladribine in a DCK-dependent and independent manner (corresponding to Figure 6c). Multiple comparisons were performed with Dunnett's post-hoc test, taking as reference the basal differentiated condition.

**Contrast**	**Estimate**	**p-value**
Undifferentiated - Differentiated	0.2588	0.0004
0.002 μM CDB - Differentiated	-0.0923	0.4006
0.02 μM CDB - Differentiated	-0.0335	0.9591
DEO - Differentiated	0.0373	0.9244
0.002 μM CDB+DEO - Differentiated	0.0348	0.9370
0.02 μM CDB +DEO - Differentiated	-0.0895	0.4747

CDB: cladribine; DEO: deoxycytidine

Supplemental Table S29 – Effect size and p-value of Fold-change in mortality (respect to basal) of SH-SY5Y cells exposed to cladribine in a DCK-dependent and independent manner (corresponding to Figure 7a). Multiple comparisons were performed with Dunnett's post-hoc test, taking as reference the basal condition.

**Contrast**	**Estimate**	**p-value**
0.002 μM CDB - Basal	-0.03463	0.9959
0.02 μM CDB - Basal	0.08924	0.8335
DEO - Basal	0.2609	0.0803
0.002 μM CDB+DEO - Basal	0.2228	0.1528
0.02 μM CDB +DEO - Basal	0.2128	0.1798

CDB: cladribine; DEO: deoxycytidine

Supplemental Table S30 – Effect size and p-value of % LDH release cytokine-challenged SH-SY5Y cells exposed to cladribine in a DCK-dependent and independent manner (corresponding to Figure 7b). Multiple comparisons were performed with Dunnett's post-hoc test, taking as reference the stimulated condition.

Contrast	Estimate	p-value
Basal - Stimulated	-16.9417	<0.0001
0.002 μM CDB - Stimulated	2.0304	0.8207
0.02 μM CDB - Stimulated	-1.6785	0.9070
DEO - Stimulated	1.6771	0.9073
0.002 μM CDB+DEO - Stimulated	3.5599	0.3658
0.02 μM CDB +DEO - Stimulated	-0.1281	1.0000

CDB: cladribine; DEO: deoxycytidine

Supplemental Table S31 – Effect size and p-value of Fold-change of mortality respect to basal in TBHP-challenged SH-SY5Y cells exposed to cladribine in a DCK-dependent and independent manner (corresponding to Figure 7c). Multiple comparisons were p erformed with Dunnett's post-hoc test, taking as reference the TBHP-stimulated condition.

Contrast	ODDS ratio	p-value
Basal – TBHP-stimulated	0.071	<0.0001
0.002 μM CDB - TBHP-stimulated	0.326	<0.0001
0.02 μM CDB - TBHP-stimulated	0.321	<0.0001
DEO - TBHP-stimulated	1.155	0.5708
0.002 μM CDB+DEO - TBHP-stimulated	0.343	<0.0001
0.02 μM CDB +DEO - TBHP-stimulated	0.238	<0.0001

CDB: cladribine; DEO: deoxycytidine

Supplemental Table S32 – Effect size and p-value of circularity in glutamate-challenged SH-SY5Y cells exposed to cladribine in a DCK-dependent and independent manner (corresponding to Figure 7e). Multiple comparisons were performed with Dunnett's post-hoc test, taking as reference the Glutamate-stimulated condition.

Contrast	Estimate	p-value
Basal – Glutamate-stimulated	-0.13859	0.0015
0.002 μM CDB - Glutamate-stimulated	0.00912	0.9950
0.02 μM CDB - Glutamate-stimulated	0.00992	0.9926
DEO - Glutamate-stimulated	-0.00101	1.0000
0.002 μM CDB+DEO - Glutamate-stimulated	-0.02235	0.9242
0.02 μM CDB +DEO - Glutamate-stimulated	0.00467	0.9992

CDB: cladribine; DEO: deoxycytidine

Supplemental Table S33 – Effect size and p-value of cell area in glutama te-challenged SH-SY5Y cells exposed to cladribine in a DCK-dependent and independent manner (corresponding to Figure 7f). Multiple comparisons were performed with Dunnett's post-hoc test, taking as reference the Glutamate-stimula ted condition.

**Contrast**	**Estimate**	**p-value**
Basal – Glutamate-stimulated	0.42762	<0.0001
0.002 μM CDB - Glutamate-stimulated	0.00594	0.9997
0.02 μM CDB - Glutamate-stimulated	-0.00268	1.0000
DEO - Glutamate-stimulated	-0.01904	0.9888
0.002 μM CDB+DEO - Glutamate-stimulated	0.04897	0.8441
0.02 μM CDB +DEO - Glutamate-stimulated	-0.03272	0.9472

CDB: cladribine; DEO: deoxycytidine

Supplemental Table S34 – Effect size and p-value of aspect ratio in glutama te-challenged SH-SY5Y cells exposed to cladribine in a DCK-dependent and independent manner (corresponding to Figure 7g). Multiple comparisons were performed with Dunnett's post-hoc test, taking as reference the Glutamate-stimulated condition.

**Contrast**	**Estimate**	**p-value**
Basal – Glutamate-stimulated	0.0995	<0.0001
0.002 μM CDB - Glutamate-stimulated	-0.0569	0.0365
0.02 μM CDB - Glutamate-stimulated	-0.0535	0.0394
DEO - Glutamate-stimulated	-0.0283	0.4756
0.002 μM CDB+DEO - Glutamate-stimulated	-0.0146	0.8831
0.02 μM CDB +DEO - Glutamate-stimulated	-0.0358	0.2658

CDB: cladribine; DEO: deoxycytidine

Supplemental Table S35 – Effect size and p-value of % LDH release cytokine-challenged MO3.13 cells exposed to cladribine in a DCK-dependent and independent manner (corresponding to Figure 8a). Multiple comparisons were performed with Dunnett's post-hoc test, taking as reference the cytokine-stimulated condition.

**Contrast**	**Estimate**	**p-value**
Basal – cytokine-stimulated	-21.0214	0.0051
0.002 μM CDB - cytokine-stimulated	-0.8358	1.0000
0.02 μM CDB - cytokine-stimulated	2.7329	0.9823
DEO - cytokine-stimulated	-4.8191	0.8274
0.002 μM CDB+DEO - cytokine-stimulated	0.05619	1.0000
0.02 μM CDB +DEO - cytokine-stimulated	-4.2989	0.8826

CDB: cladribine; DEO: deoxycytidine

Supplemental Table S36 – Effect size and p-value of % LDH release H_2O_2-challenged MO3.13 cells exposed to cladribine in a DCK-dependent and independent manner (corresponding to Figure 8a). Multiple comparisons were performed with Dunnett's post-hoc test, taking as reference the H_2O_2-stimulated condition.

**Contrast**	**Estimate**	**p-value**
Basal – H_2O_2-stimulated	-50.8678	<0.0001
0.002 μM CDB - H_2O_2-stimulated	9.0341	0.0678
0.02 μM CDB - H_2O_2-stimulated	6.7324	0.2203
DEO - H_2O_2-stimulated	3.0585	0.8457
0.002 μM CDB+DEO - H_2O_2-stimulated	4.7879	0.5104
0.02 μM CDB +DEO - H_2O_2-stimulated	2.7864	0.8881

CDB: cladribine; DEO: deoxycytidine
